# Survey of quality of life, phenotypic expression, and response to treatment in Krabbe leukodystrophy

**DOI:** 10.1002/jmd2.12033

**Published:** 2019-04-11

**Authors:** Thomas J. Langan, Amy Barczykowski, Kabir Jalal, Laura Sherwood, Heather Allewelt, Joanne Kurtzberg, Randy L. Carter

**Affiliations:** ^1^ Department of Neurology, School of Medicine and Biomedical Sciences University at Buffalo Buffalo New York; ^2^ Department of Biostatistics, Population Health Observatory, School of Public Health and Health Professions University at Buffalo Buffalo New York; ^3^ Department of Pediatrics Duke University School of Medicine Durham North Carolina

**Keywords:** neurodegeneration, newborn screening, transplantation

## Abstract

**Objectives:**

To develop a quality of life (QOL) survey for Krabbe disease (KD), and to thereby improve understanding of its phenotypic expression and response to treatment.

**Methods:**

The survey, the Leukodystrophy Quality of Life Assessment (LQLA) and the Vineland Adaptive Behavior Scales were co‐administered to 33 patients or their caretakers. These included the phenotypes of early infantile KD (EIKD; 0‐6 months old at onset), late infantile cases (LIKD; 7‐12 months old at onset), and cases that emerged after 12 months old, late onset (LOKD). The sample included cases with and without stem cell transplantation (SCT). Reliability and concurrent validity were assessed for overall and subscale scores. Analysis of variance tested differences in QOL between phenotypes and transplant groups (none, pre‐, post‐symptom).

**Results:**

Good concurrent validity with the Vineland was shown for total, communication, daily activity, social, and motor scales and good reliability was observed. LOKD cases had better communication skills than either EIKD or LIKD and better overall QOL than EIKD. Analyses of individual items showed that communication items, mostly, contributed significantly to phenotype differences. Presymptomatic SCT significantly improved QOL compared to postsymptomatic SCT or no treatment. Presymptomatically treated patients had near‐normal total scores.

**Conclusions:**

The LQLA is valid and reliable. Despite small sample size, phenotypic demarcation was determined to be due mainly to differences in communication skills. There was a relative enhancement of QOL in LOKD patients, and in those who had presymptomatic SCT. These results apply to the current controversy about recommendations for newborn screening for this condition.

**Synopsis**

A recently developed quality of life survey identifies quantitative differences between Krabbe disease phenotypes and provides new evidence of the benefit of presymptomatic stem cell transplantation in this condition.

## 1 INTRODUCTION

Knud Krabbe first described the condition that bears his name over 100 years ago.^1^ Krabbe disease (KD), or globoid cell leukodystrophy (OMIM#245200), was, over the ensuing century, established as a severe, invariably fatal neurologic disorder affecting mainly infants and children.^2^, ^3^, ^4^, ^5^ Yet the natural history and phenotypic progression of this disorder remain incompletely understood.^2^, ^6^, ^7^ All cases appear to be caused by an autosomal recessive deficiency of the enzyme galacto‐cerebrosidase (GALC),^8^, ^9^ but genotype/phenotype correlation remains poorly elucidated, except for homozygous expression of the 30 kb deletion that predicts early infantile onset Krabbe disease (EIKD).^2^, ^4^, ^10^ Current descriptions of KD phenotype focus upon age of onset, distinguishing EIKD from later onset variants that can emerge in later infancy, childhood, or adulthood.^2^, ^4^, ^5^, ^6^ But the age demarcations and symptomatic characterization of the later onset variants, as well as their relative proportions, are not defined unequivocally.^7^

The only currently available therapy for KD is stem cell transplantation (SCT), which, while not curative, modifies phenotype optimally only if used before symptoms develop.^11^, ^12^, ^13^ However, because newborn screening (NBS) for KD has not been widely adopted,^14^ most cases of EIKD currently present with at least mild symptoms. Virtually all late onset cases are identified only after symptoms develop.^6^, ^7^ The need to better define natural history to determine when treatment will be effective is a major challenge not only in KD^7^, ^13^, ^15^, ^16^, ^17^, ^18^ but also more generally in childhood leukodystrophies.^19^

We report here our utilization of the World Wide Registry (WWR), a data resource that contains information on almost 200 affected or at‐risk cases^2^, ^6^, ^20^, ^21^ to better define the phenotypes that comprise the natural history of KD variants. The WWR facilitates study of this rare condition.^2^, ^6^, ^20^, ^21^ Analysis of death certificates suggested a frequency of EIKD of 1 in 244 000 and later onset cases are even more rare.^22^ However, the New York State experience after 10 years of NBS for Krabbe^18^ identified only five definite cases among over 2 million screened.

The need for a broad exploration of quality of life (QOL) as an expression of phenotype became apparent during the course our recent analysis of the effect of SCT upon survival in symptomatic KD patients.^21^ QOL is indeed considered a critical determinant of the response to therapy in KD.^23^ Consequently, we conducted phone surveys of currently surviving patients enrolled in the registry.

## 2 METHODS

### 2.1 Identification of KD patients

The WWR consists of de‐identified data regarding 198 patients who have been diagnosed with KD, or who, by virtue of concerning genotype or diminished GalC enzyme level, are considered to be at high risk for developing symptoms of KD.^2^, ^6^, ^21^ The de‐identified data are maintained in the Longitudinal Pediatric Data Resource of the Newborn Screening Translation Research Network.^24^, ^25^ Amongst the 198 registrants, 45 were determined as likely to be living at the onset of this study, based upon our periodic follow‐up calls and information provided by advocacy groups. We were able to successfully reach 33 of these surviving individuals or family members for telephone interviews.

We classified phenotypes according to age of onset.^2^, ^4^, ^6^, ^21^ Thus, EIKD was considered to emerge between birth and 6 months, and late infantile KD (LIKD) between 7 months and 1 year. Because of the limited sample size, the current analysis pooled those cases presenting after 1 year of life as late onset KD (LOKD), merging as a single group of cases which could be identified as juvenile and adult onset.^2^, ^6^ Alternative classifications of this very rare^22^ disorder have been advanced. Earlier investigators, for example, suggested that LIKD might emerge as late as age 3 or 4 years.^7^, ^26^ But for the current investigation, we rely on the classification that is supported by earlier analysis of data contained in the WWR.^2^, ^6^

The current data set includes 11 EIKD cases, seven LIKD cases, 12 LOKD cases, and three registrants who remain asymptomatic despite genotypes indicating risk for LOKD.^10^ SCT was achieved in cases of EIKD (two before and two after symptoms emerged), LIKD (one after symptoms manifested) and LOKD (three before and three after symptoms appeared). Distinguishing whether cases were either symptomatic or presymptomatic for treatment was accomplished by examining medical records contained in the WWR. However, we acknowledge that the presymptomatic patients may have experienced subtle or early complaints (eg, irritability and clumsiness) that may presage more definite neurologic symptoms (including developmental regression, spasticity and blindness) in the infantile^2^ and late onset^6^ phenotypes. All patients who underwent SCT had documented abnormalities in neurophysiologic and neuroimaging tests prior to the procedure, which preceded development of clinical symptoms. In addition, these patients also had a family history of a sibling with identical mutations who developed and died from symptomatic KD during early childhood.

### 2.2 Development and validation of the survey

QOL has been described as a fundamental component of phenotype in genetic disorders generally,^27^ and specifically in lysosomal disorders and leukodystrophies, the class of disorders that includes KD.^28^, ^29^, ^30^

Proprietary QOL questionnaires have been applied to various pediatric illnesses, for example, sickle cell disease,^31^ inflammatory bowel disease,^32^ dermatologic conditions,^33^ epilepsy,^34^ autism,^35^ and head injuries.^36^ When considered together, these studies indicate that QOL surveys should be modified for application to specific ages and disease entities.^31^, ^36^, ^37^ Even with such targeting, however, some survey recipients have expressed dissatisfaction with relevance of the questions to their life situations.^36^

The Vineland Adaptive Behavior Scales have been demonstrated to have utility in assessing QOL in significantly disabled individuals.^38^, ^39^, ^40^ However, like other QOL metrics, the relevance of questions to leukodystrophy patients and families and its extended length makes the Vineland unsuitable for use in this population.

We consequently devised a new survey, named the Leukodystrophy Quality of Life Assessment (LQLA—see Data S1), which includes questions relevant to the extreme burden of disability and medical problems experienced by most surviving KD patients.^2^, ^4^, ^6^, ^21^ Our intent was to construct an open‐access, freely available questionnaire suitable for very disabled individuals (from infancy to adulthood) with a leukodystrophy or similar disorder that could be administered in less than 30 minutes. Some of the specific questions were culled from earlier preliminary surveys developed at the respective institutions of the current authors. The survey is appended. It contains questions about social, communicative, and motor abilities, caretaker satisfaction, and medical and neurological symptoms.

To confirm its validity, the LQLA was compared to the Vineland. Like the Vineland, the LQLA was constructed to provide an overall score, as well as subscores in the communication, daily living, social, and motor domains. Both the LQLA and the Vineland were administered to each of the 33 WWR registrants, or caretakers of nonverbal registrants, whom were reached by phone.

### 2.3 Statistical analyses

The LQLA was assessed for concurrent validity with the Vineland using Pearson correlation analysis. To evaluate internal consistency within the LQLA, Cronbach's alpha (which increases with increasing average inter‐item correlation) was computed.^41^ Concurrent validity and internal consistency were examined for all patients with, or at high risk of, KD (N = 33). This maximized the sample size used for the comparison.

Analysis of variance (ANOVA) was utilized to identify overall differences between phenotype (EIKD, LIKD, LOKD), and transplant (no transplant, postsymptomatic, presymptomatic) groups. Duncan's multiple range test was used to identify pairwise differences between phenotypic and transplant groups. ANOVA and Duncan's multiple range test was performed for the sample of Krabbe cases (N = 30).

Pearson correlations, Cronbach's alpha, ANOVA and Duncan's multiple range test were performed for the overall survey results and each of the four subscales.

Finally, Fisher's exact test was used to identify specific survey items that showed phenotypic differences, with the Benjamini‐Hochberg procedure applied to adjust for multiple comparisons. Differences between the standard Bonferroni method of controlling the false positive rate, which can suppress true positive effects, and the Benjamini‐Hochberg test which limits alpha error inflation or the false discovery rate (chosen here to be *α* = 0.25) are well described.^42^ Fisher's exact test was restricted to the sample of Krabbe cases that were not transplanted presymptomatically (N = 25).

The questions of the LQLA were arranged in domains that parallel those of the Vineland; there was an overall score, as well as subscores in the communication, daily living, family/social and motor subscales. Each item was scored as a 0 or 1, where 0 indicated a negative response and 1 indicated a positive response. Positive responses were determined based on the choice indicating the closest proximity to the best QOL. Items regarding speaking words and putting words together were assigned a 0 only if the patient was nonverbal. For the LQLA, the maximum overall score was 41, and maximum subscale scores were respectively 10, 14, 11 and 6 for the communication, daily living, family/social and motor subscales.

## 3 RESULTS

The registrants or caretakers were successfully administered both the LQLA and the Vineland over the course of a single telephone call. The LQLA and Vineland scales were administered, respectively, over periods of 10 to 15 minutes and 15 to 30 minutes. The respondents were told at the onset of each interview that administration of the two questionnaires could be achieved over more than a single call if this was preferable. However, all interviews were completed in a single call.

Correlations (*R* ^2^) between the LQLA and the Vineland total and subscale scores are shown in Table 1. Values close to 1 suggest near‐perfect validity. Good to excellent concurrent validity (*R* ^2^ range: 0.5923‐0.9522) was demonstrated for the overall scores of the two surveys, and also for the communication, daily living, and motor subdomains. Fair validity was demonstrated for the group of queries assessing social skills (*R* ^2^ = 0.4975). These results suggest that the LQLA provides additional information beyond the Vineland while maintaining a substantial degree of validity against the Vineland.

**Table 1:** Validity and reliability of LQLA

Full survey sample (N = 33)^a^		
Measure	*R* ^2^	Cronbach's alpha (raw)
Overall	0.8631	0.9525
Communication	0.5923	0.9118
Daily living	0.7621	0.8950
Social	0.4975	0.7503
Motor	0.9522	0.9418

a Age of participants at time of survey (months): 14.5, 29.0, 44.0, 52.0, 65.0, 65.6, 66.3, 67.3, 70.4, 73.9, 77.7, 78.3, 82.1, 92.8, 133.6, 143.5, 148.6, 167.5, 175.1, 186.6, 196.8, 197.1, 208.9, 238.1, 314.4, 373.6, 390.3, 447.8, 451.6, 456.2, 460.1, 645.7, and 664.0.

Table 1 also includes examination of the reliability/internal consistency of the LQLA using Cronbach alpha values, with values near 1 suggesting strong reliability. Table 1 shows that the overall score and the scores for the communication, daily living, and motor subscores reflected excellent reliability. The social scales demonstrated good reliability.

Having developed a valid and reliable survey for determining QOL in KD patients, we next evaluated the capacity of the LQLA to identify differences amongst the phenotypic groups based upon age of onset. This analysis, shown in Table 2, included only the 25 symptomatic KD patients. For each domain, Table 2 provides means by phenotype and *P*‐values assessing overall differences between phenotype. Table 2 also contains groupings for Duncan's multiple range test. A superscript of “a” indicates the LO mean is higher than the EIKD; “b” indicates the LO mean is higher than the LIKD mean; “c” indicates no significant phenotypic differences. Because of the small sample size, the EIKD cases were compared to the combined late infantile and later onset cases. Significant differences in QOL were shown between these groups. In terms of the overall and communication scores, the later onset cases had substantially better QOL than those with early infantile onset. Differences in the other QOL domains were not significant among these two age‐of‐onset groups (Table 2).

**Table 2:** Phenotypic differences

Sample of Krabbe cases (N = 30)					
Measure	Phenotype ANOVA *P*‐value	Estimated mean by phenotype			Duncan grouping
		EIKD (N = 11)	LIKD (N = 7)	LO (N = 12)	
Overall	.0175	13.636	17.286	24.167	a
Communication	.0028	4.1820	5.4290	8.2500	a ^,^ b
Daily living	.0645	4.818	6.5710	8.3330	a
Social	.6446	3.8182	3.8571	4.5833	c
Motor	.0369	0.8182	1.4286	3.0000	a ^,^ b

a Indicates LO mean significantly higher than EIKD mean.

b Indicates LO mean significantly higher than LIKD mean.

c Indicates no significant differences between group means.

We next considered the critical question of the effect of SCT upon QOL.^23^ Here we used the LQLA to determine QOL differences between affected patients transplanted before or after the start of symptoms, and those who were never treated with SCT. These results are summarized in Table 3. For each domain, Table 3 provides means by phenotype and *P*‐values assessing overall differences between transplant times. For Duncan's multiple range test, a superscript of “a” indicates the presymptomatic transplant group mean is higher than the nontransplant group, and “b” indicates the presymptomatic mean is higher than the postsymptomatic mean. The enhancement of QOL by application of SCT before emergence of symptoms was striking. Presymptomatic transplantation resulted in significant and quantifiable enhancement of QOL compared with either postsymptom or nontransplanted patients in overall scores (82% of maximal scores) and each of the four subscales. In transplanted patients, communication, daily living, social/family and motor scores were respectively 96%, 86%, 40% and 83% of maximum QOL scores. This suggests that while transplanted patients score highly in most QOL domains, the social/family domain may be resistant to QOL improvements brought on by successful transplant.

**Table 3:** Differences between transplant groups

Sample of Krabbe cases (N = 30)					
Measure	Transplant ANOVA *P*‐value	Estimated mean by transplant time			Duncan grouping
		None (N = 19)	Postsymptom (N = 6)	Presymptom (N = 5)	
Overall	.0029	15.526	17.167	32.6000	a ^,^ b
Communication	.0178	5.0530	6.5000	9.6000	a ^,^ b
Daily living	.0142	5.5000	5.789	11.2000	a ^,^ b
Social	.0219	3.526	3.833	6.8000	a ^,^ b
Motor	.0042	1.1580	1.3333	5.0000	a ^,^ b

a Indicates presymptomatic transplant mean significantly higher than no‐transplant mean.

b Indicates presymptomatic transplant mean significantly higher than postsymptomatic transplant mean.

Because we report here development of a new survey designed to determine QOL in infants and children severely affected with neurologic disability, we considered it of interest to determine which items of the survey contribute most to identification of differences between the phenotypic subgroups. This analysis is shown in Table 4, with low *P*‐values suggesting differences between the phenotypes. Shown there is a ranked order of significance for the seven questions that accounted best for differences in QOL between the early infantile, late infantile and late onset phenotypic groups. These groups were distinguished between each other most prominently by items that are related to communication. Only one of the seven items, the capacity to roll over independently, can be considered to represent an exclusively motor ability.

**Table 4:** LQLA items significantly associated with phenotype

Fisher's exact test for item response by phenotype excluding presymptomatic transplants (N = 25)				
	Percentage of negative responses by phenotype			
Survey item	EIKD (N = 9)	LIKD (N = 7)	LO (N = 9)	Phenotype Fisher *P*‐value
Communicates needs	77.78	51.14	0	.0019
Attends school	75	42.86	0	.0030
Babbles	100	42.86	33.33	.0056
Speaks words	88.89	71.43	22.22	.0149
Rolls over	100	71.43	44.44	.0237
General communication	100	71.43	44.44	.0237
Smiles	66.67	14.29	11.11	.0403

## 4 DISCUSSION

These results establish the LQLA as a valid and reliable survey for determining QOL in KD (Table 1), and presumably also for any disorder which causes a similar impairment of neurologic function. It may consequently find use in future research that examines benefits of therapies for neurodegenerative disorders, in which QOL is indeed recognized as an important outcome of treatment.^28^, ^29^, ^30^ It is possible that the survey could be shortened to include the items contributing most to the KD phenotypic difference (Table 4). However, determination of its final iteration will require testing more broadly in KD and other neurodegenerative illnesses.

We acknowledge that the major shortcoming of the current study is the small sample size. It must, however, also be acknowledged that KD is extremely rare.^18^, ^22^ The possibility of reaching 30 living, affected patients for phone interviews, made possible by their registration in the WWR, provided an opportunity to better understand phenotypic expression of this very rare disorder.

The findings above (Table 2), which compare QOL in time of onset‐based groups (EIKD and later onset cases), provide new, quantitative distinctions between these phenotypes. The natural history of EIKD, therefore, can be concluded to involve certain identifiable components of QOL more prominently than in patients who experience later onset of symptoms. However, the QOL differences between phenotype were not observed on the social domain. This may indicate that social experiences are common between phenotypes.

This line of inquiry was continued by examination of the specific contribution of types of survey questions that contributed to the differences between ages of onset‐based phenotypes (Table 4). The greatest differences between these phenotypes were attributable to communication skills. KD primarily affects the white matter of the brain and peripheral nerves, parts of the nervous system that are not primarily associated with neurocognitive capacities, but cognition may be affected prominently in the early stages of the disorder.^2^, ^4^, ^6^ KD indeed results in extensive involvement of white matter tracts but also atrophy of cerebral gray matter.^43^, ^44^

In addition, the current findings are consistent with a recent report by others identifying outcomes of SCT in KD,^13^ where it was found that the treatment specifically preserves cognitive abilities and results in improvement of receptive language skills, even as motor disability may advance in treated patients.^13^ These results are concordant with the current finding that more severely affected patients have diminished QOL in the realm of communication skills (Table 4).

The benefit of SCT for KD was also addressed by the current study of QOL. The enhancement of overall QOL survey scores, as well as of scores in each of the individual communication, daily living, social and motor domains, provide new, strikingly significant proof of the benefit of presymptomatic transplantation (Table 3).

However, an alternative explanation for the apparent benefit of presymptomatic transplantation would be that these patients were incorrectly diagnosed prior to treatment; that they represented either false positives or possible later onset cases who were not destined to develop symptoms. While this possibility cannot be absolutely excluded, it is extraordinarily unlikely. Selection of candidates for transplantation is based upon several parameters, including family history, enzyme activity, genotype, and the presence of active, if presymptomatic disease on neurophysiologic and neuroimaging testing prior to transplantation.^11^, ^12^, ^13^

The current and recently published results^13^ regarding outcomes of SCT may have important implications for Krabbe NBS. Thus, even though screening for KD is now conducted in a few states,^14^ this condition was rejected for inclusion in the recommended universal NBS panel in the United States. Reasons cited for this rejection included insufficient knowledge about KD in three specific areas: the benefit of SCT, the need for a better understanding of the EIKD phenotype, and for an improved approach to diagnosis.^45^

SCT enhances duration of survival in KD.^11^, ^21^ But unequivocal improvement in specific symptoms remained an open question until recent research into the outcomes of transplanted patients.^13^ The current results (Table 3) confirm quantifiable QOL improvement in presymptomatically transplanted patients. Hence, the gap of information regarding the benefit of SCT that was cited in rejection of Krabbe for universal screening^45^ has now been substantially filled.

The data shown in Tables 2 and 4 that compared EIKD cases with those having later onset indicated that among aspects of QOL that were surveyed, those specifically related to communication skills accounted for phenotypic differences. The results of this article, therefore, improve phenotypic definition of EIKD.

The final area of incomplete information that precluded universal NBS for KD^45^ is that of accurate and rapid diagnosis. Because of the initially high false positive rate and low positive predictive value of the Krabbe NBS protocol developed in New York state,^14^, ^18^ enthusiasm for NBS for KD was limited. However, learning from this initial experience and recent approaches using biomarkers of infantile KD suggest that rapid diagnosis of the newborn with early infantile disease is possible using a combination of NBS assays, enzyme and psychosine levels.^13^, ^15^, ^17^, ^46^ The results presented here, on the one hand, provide a clearer picture of the phenotypic expression of KD, and of the response of affected patients to current treatment. They may, on the other hand, become part of a broader body of the experimental evidence used to establish the feasibility of NBS for this dreadful affliction of children in the future.

## CONFLICT OF INTERESTS

All authors are without conflict of interest.

The authors confirm independence from the sponsors; the content of the article has not been influenced by the sponsors.

## AUTHOR CONTRIBUTIONS

T.J.L.: Study design, methodology, investigation, oversight of study activities, funding acquisition, data curation, formal analysis, drafting and editing of manuscript. A.B.: Study design, methodology, investigation, data curation, drafting and editing of manuscript. R.L.C.: Study design, methodology, investigation, funding acquisition, formal analysis, drafting and editing of manuscript. K.J.: Study design, methodology, investigation, funding acquisition, data curation, formal analysis, drafting and editing of manuscript. J.K.: Study design, methodology, investigation, resources, editing of manuscript. H.A.: Study design, methodology, investigation, resources, editing of manuscript. L.S.: Study design, methodology, investigation, editing of manuscript.

## GUARANTOR

Dr. Thomas J. Langan.

## ETHICAL APPROVAL STATEMENT

This study was approved by the University at Buffalo Institutional Review Board.

## MATERIAL AVAILABILITY

The de‐identified data are maintained in the Longitudinal Pediatric Data Resource (LPDR) of the Newborn Screening Translation Research Network (NBSTRN).

## Supporting information


**Data S1.** Leukodystrophy Quality of Life Assessment (LQLA).

## References

[1] Krabbe K . A new familial, infantile form of diffuse brain‐sclerosis. Brain. 1916;39:74‐114. 10.1093/brain/awt232 24137751

[2] Duffner PK , Barczykowski A , Jalal K , Yan L , Kay DM , Carter RL . Early infantile Krabbe disease: results of the world‐wide Krabbe registry. Pediatr Neurol. 2011;45:141‐148. 21824559 10.1016/j.pediatrneurol.2011.05.007

[3] Hagberg B , Sourander P , Svennerholm L . Diagnosis of Krabbe's infantile leucodystrophy. J Neurol Neurosurg Psychiatry. 1963;26:195‐198. 13951860 10.1136/jnnp.26.3.195 PMC1074213

[4] Wenger DA , Luzi P , Rafi MA . Krabbe disease: are certain mutations disease‐causing only when specific polymorphisms are present or when inherited in trans with specific second mutations? Mol Genet Metab. 2014;111:307‐308. 24388568 10.1016/j.ymgme.2013.12.009

[5] Wenger DA , Rafi MA , Luzi P . Krabbe disease: one hundred years from the bedside to the bench to the bedside. J Neurosci Res. 2016;94:982‐989. 27638583 10.1002/jnr.23743

[6] Duffner PK , Barczykowski A , Kay DM . Later onset phenotypes of Krabbe disease: results of the world‐wide registry. Pediatr Neurol. 2012b;46:298‐306. 22520351 10.1016/j.pediatrneurol.2012.02.023

[7] Sakai N , Otomo T . Challenge of phenotype estimation for optimal treatment of Krabbe disease. J Neurosci Res. 2016;94:1025‐1030. 27638587 10.1002/jnr.23914

[8] Suzuki K . Biochemical pathogenesis of genetic leukodystrophies: comparison of metachromatic leukodystrophy and globoid cell leukodystrophy (Krabbe's disease). Neuropediatrics. 1984;15(Suppl):32‐36. 6152811 10.1055/s-2008-1052380

[9] Suzuki K , Suzuki Y . Globoid cell leucodystrophy (Krabbe's disease): deficiency of galactocerebroside beta‐galactosidase. Proc Natl Acad Sci U S A. 1970;66:302‐309. 5271165 10.1073/pnas.66.2.302 PMC283044

[10] Saavedra‐Matiz CA , Luzi P , Nichols M , Orsini JJ , Caggana M , Wenger DA . Expression of individual mutations and haplotypes in the galactocerebrosidase gene identified by the newborn screening program in New York state and in confirmed cases of Krabbe's disease. J Neurosci Res. 2016;94:1076‐1083. 27638593 10.1002/jnr.23905

[11] Escolar ML , Poe MD , Provenzale JM . Transplantation of umbilical‐cord blood in babies with infantile Krabbe's disease. N Engl J Med. 2005;352:2069‐2081. 15901860 10.1056/NEJMoa042604

[12] Musolino PL , Lund TC , Pan J . Hematopoietic stem cell transplantation in the leukodystrophies: a systematic review of the literature. Neuropediatrics. 2014;45:169‐174. 24459069 10.1055/s-0033-1364179 PMC4157669

[13] Wright MD , Poe MD , DeRenzo A , Haldal S , Escolar ML . Developmental outcomes of cord blood transplantation for Krabbe disease: a 15‐year study. Neurology. 2017;89:1365‐1372. 28855403 10.1212/WNL.0000000000004418 PMC5649761

[14] Orsini JJ , Saavedra‐Matiz CA , Gelb MH , Caggana M . Newborn screening for Krabbe's disease. J Neurosci Res. 2016;94:1063‐1075. 27638592 10.1002/jnr.23781 PMC5328187

[15] Carter RL , Wrabetz L , Jalal K . Can psychosine and galactocerebrosidase activity predict early‐infantile Krabbe's disease presymptomatically? J Neurosci Res. 2016;94:1084‐1093. 27638594 10.1002/jnr.23793

[16] Escolar ML , Jones SA , Shapiro EG , Horovitz DDG , Lampe C , Amartino H . Practical management of behavioral problems in mucopolysaccharidoses disorders. Mol Genet Metab. 2017;122S:35‐40. 29170079 10.1016/j.ymgme.2017.09.010

[17] Turgeon CT , Orsini JJ , Sanders KA . Measurement of psychosine in dried blood spots—a possible improvement to newborn screening programs for Krabbe disease. J Inherit Metab Dis. 2015;38:923‐929. 25762404 10.1007/s10545-015-9822-z

[18] Wasserstein MP , Andriola M , Arnold G , et al. Clinical outcomes of children with abnormal newborn screening results for Krabbe disease in New York state. Genet Med. 2016;18:1235‐1243. 27171547 10.1038/gim.2016.35

[19] Osorio MJ , Goldman SA . Glial progenitor cell‐based treatment of the childhood leukodystrophies. Exp Neurol. 2016;283:476‐488. 27170209 10.1016/j.expneurol.2016.05.010 PMC5340082

[20] Duffner PK , Granger C , Lyon N . Developmental and functional outcomes in children with a positive newborn screen for Krabbe disease: a pilot study of a phone‐based interview surveillance technique. J Pediatr. 2012a;161:258‐263. 22381022 10.1016/j.jpeds.2012.01.044

[21] Langan TJ , Barcykowski AL , Dare J , Pannullo EC , Muscarella L , Carter RL . Evidence for improved survival in postsymptomatic stem cell‐transplanted patients with Krabbe's disease. J Neurosci Res. 2016;94:1189‐1194. 27638603 10.1002/jnr.23787 PMC5484586

[22] Barczykowski AL , Foss AH , Duffner PK , Yan L , Carter RL . Death rates in the U.S. due to Krabbe disease and related leukodystrophy and lysosomal storage diseases. Am J Med Genet A. 2012;158A:2835‐2842. 22991292 10.1002/ajmg.a.35624

[23] Krivit W , Lockman LA , Watkins PA , Hirsch J , Shapiro EG . The future for treatment by bone marrow transplantation for adrenoleukodystrophy, metachromatic leukodystrophy, globoid cell leukodystrophy and Hurler syndrome. J Inherit Metab Dis. 1995;18:398‐412. 7494399 10.1007/BF00710052

[24] Botkin JR , Lewis MH , Watson MS . Parental permission for pilot newborn screening research: guidelines from the NBSTRN. Pediatrics. 2014;133:e410‐e417. 24394680 10.1542/peds.2013-2271 PMC3904278

[25] Urv TK , Parisi MA . Newborn screening: beyond the spot. Adv Exp Med Biol. 2017;1031:323‐346. 29214581 10.1007/978-3-319-67144-4_19

[26] Loonen MC , Van Diggelen OP , Janse HC , Kleijer WJ , Arts WF . Late‐onset globoid cell leucodystrophy (Krabbe's disease). Clinical and genetic delineation of two forms and their relation to the early‐infantile form. Neuropediatrics. 1985;16:137‐142. 4047347 10.1055/s-2008-1052558

[27] Edwards JG , Feldman G , Goldberg J . Expanded carrier screening in reproductive medicine‐points to consider: a joint statement of the American College of Medical Genetics and Genomics, American College of Obstetricians and Gynecologists, National Society of Genetic Counselors, Perinatal Quality Foundation, and Society for Maternal‐Fetal Medicine. Obstet Gynecol. 2015;125:653‐662. 25730230 10.1097/AOG.0000000000000666

[28] Grabowski GA . Phenotype, diagnosis, and treatment of Gaucher's disease. Lancet. 2008;372:1263‐1271. 19094956 10.1016/S0140-6736(08)61522-6

[29] Peters C , Steward CG , National Marrow Donor P, International Bone Marrow Transplant R, Working Party on Inborn Errors EBMTG . Hematopoietic cell transplantation for inherited metabolic diseases: an overview of outcomes and practice guidelines. Bone Marrow Transplant. 2003;31:229‐239. 12621457 10.1038/sj.bmt.1703839

[30] Biffi A , Lucchini G , Rovelli A , Sessa M . Metachromatic leukodystrophy: an overview of current and prospective treatments. Bone Marrow Transpl. 2008;42:S2. 10.1038/bmt.2008.275 18978739

[31] Panepinto JA , Paul Scott J , Badaki‐Makun O , et al. Determining the longitudinal validity and meaningful differences in HRQL of the PedsQL sickle cell disease module. Health Qual Life Outcomes. 2017;15:124. 28606098 10.1186/s12955-017-0700-2 PMC5468970

[32] Watson KL Jr , Kim SC , Boyle BM , Saps M . Prevalence and impact of functional abdominal pain disorders in children with inflammatory bowel diseases (IBD‐FAPD). J Pediatr Gastroenterol Nutr. 2017;65:212‐217. 27906801 10.1097/MPG.0000000000001479

[33] Blome C , Radtke MA , Eissing L , Augustin M . Quality of life in patients with atopic dermatitis: disease burden, measurement, and treatment benefit. Am J Clin Dermatol. 2016;17:163‐169. 26818063 10.1007/s40257-015-0171-3

[34] Lai JS , Nowinski CJ , Zelko F . Validation of the Neuro‐QoL measurement system in children with epilepsy. Epilepsy Behav. 2015;46:209‐214. 25862469 10.1016/j.yebeh.2015.02.038 PMC4458416

[35] de Vries M , Geurts H . Influence of autism traits and executive functioning on quality of life in children with an autism spectrum disorder. J Autism Dev Disord. 2015;45:2734‐2743. 25835211 10.1007/s10803-015-2438-1 PMC4553152

[36] Di Battista A , Godfrey C , Soo C , Catroppa C , Anderson V . Does what we measure matter? Quality‐of‐life defined by adolescents with brain injury. Brain Inj. 2015;29:573‐582. 25642580 10.3109/02699052.2014.989905

[37] Einaudi MA , Gire C , Loundou A , Le Coz P , Auquier P . Quality of life assessment in preterm children: physicians' knowledge, attitude, belief, practice—a KABP study. BMC Pediatr. 2013;13:58. 23601174 10.1186/1471-2431-13-58 PMC3637183

[38] Balboni G , Pedrabissi L , Molteni M , Villa S . Discriminant validity of the Vineland scales: score profiles of individuals with mental retardation and a specific disorder. Am J Ment Retard. 2001;106:162‐172. 11321607 10.1352/0895-8017(2001)106<0162:DVOTVS>2.0.CO;2

[39] Perry A , Factor DC . Psychometric validity and clinical usefulness of the F adaptive behavior scales and the AAMD adaptive behavior scale for an autistic sample. J Autism Dev Disord. 1989;19:41‐55. 2708303 10.1007/BF02212717

[40] Roszkowski MJ . Concurrent validity of the adaptive behavior scale as assessed by the Vineland social maturity scale. Am J Ment Defic. 1980;85:86‐89. 7446575

[41] Tavakol M , Dennick R . Making sense of Cronbach's alpha. Int J Med Educ. 2011;2:53‐55. 28029643 10.5116/ijme.4dfb.8dfd PMC4205511

[42] Benjamini Y , Hochberg Y . Controlling the false discovery rate: a practical and powerful approach to multiple testing. J Roy Stat Soc B. 1995;57:289‐300.

[43] Baram TZ , Goldman AM , Percy AK . Krabbe disease: specific MRI and CT findings. Neurology. 1986;36:111‐115. 3941765 10.1212/wnl.36.1.111

[44] Barone R , Bruhl K , Stoeter P , Fiumara A , Pavone L , Beck M . Clinical and neuroradiological findings in classic infantile and late‐onset globoid‐cell leukodystrophy (Krabbe disease). Am J Med Genet. 1996;63:209‐217. 8723112 10.1002/(SICI)1096-8628(19960503)63:1<209::AID-AJMG37>3.0.CO;2-Q

[45] Kemper AR , Knapp AA , Green NS , Comeau AM , Metterville DR , Perrin JM . Weighing the evidence for newborn screening for early‐infantile Krabbe disease. Genet Med. 2010;12:539‐543. 20601893 10.1097/GIM.0b013e3181e85721

[46] Langan TJ , Orsini JJ , Jalal K . Development of a newborn screening tool based on bivariate normal limits: using psychosine and galactocerebrosidase determination on dried blood spots to predict Krabbe disease. Genet Med. 2018 10.1038/s41436-018-0371-3. 30546085

