# Supplementary material for: Survey of quality of life, phenotypic expression, and response to treatment in Krabbe leukodystrophy
Source: JIMD Rep. 2019 Apr 11;47(1):47–54. doi: 10.1002/jmd2.12033 (PMC6498827; doi:10.1002/jmd2.12033)
Supplement: Supplementary file 1 — Data S1. Leukodystrophy Quality of Life Assessment (LQLA). [file JMD2-47-47-s001.pdf]

**Leukodystrophy Quality of Life Assessment (LQLA)**

**Communication:**

- Verbal-normal-4
- Verbal-abnormal speech-3
- Abnormal-uses sign language-2
- Communicative with assistive device-1
- Noncommunicative-0

Looks at your face

- Yes – 1
- No – 0

Smiles socially

- Yes – 1
- No – 0

Makes cooing noises

- Yes – 1
- No – 0

Babbles

- Yes – 1
- No – 0

Speaks words

- 0-0
- 1-3 - 1
- 4-10 - 2
- 11-15 – 3
- >15 – 4

Puts words together

- 0-0
- 2-3 - 1
- 4-5 - 2

Names objects/colors

- Yes – 1
- No – 0

Recognizes voices

- Yes – 1
- No – 0

Able to communicate needs

- Yes – 1
- No – 0

**Feeding:**

- Solely by mouth-independent-4
- Solely by mouth-requires assistance-3
- By mouth-requires tube for medications-2
- Some by mouth-requires tube to meet nutritional needs-1
- Solely via tube-0

**School if applicable**

**School attendance:**

- Yes -1
- No-0
- Not applicable due to age

**Grade level:**

- Appropriate for age (or above)-2
- Below expected for age-1
- Does not attend-0

**School performance:**

- Regular, mainstream classes-4
- Special Education-3
- IEP/504-2
- Other specialized resources-1
- Homebound due to health-0

**Symptoms Affecting Quality of Life**

Vision

- Normal-4
- Abnormal-no assistive device-3
- Abnormal-glasses or contacts-2
- Abnormal-other assistive device-1
- Blind-0

Hearing

- Normal-5
- Abnormal-no assistive device-4
- Abnormal-hearing aid(s)-3
- Abnormal-cochlear implants-2
- Abnormal-other assistive device-1
- Deaf-0

Pulmonary

- Room air-4
- Supplemental oxygen-3
- BIPAP/CPAP (no trach)-2
- Tracheostomy-1
- Tracheostomy with mechanical valve-0

Sitting

- Unsupported-1
- Only with support-0

Seizure

- Yes-0
- No-1

Spasticity

- Yes-0
- No-1

Bowel/Bladder incontinence

- Yes-0
- No-1

Aspiration

- Yes-0
- No-1
- Unknown

Scoliosis

- Yes-0
- No-1

Orthopedic surgeries

- Yes-0
- No-1
- Unknown

**Family and Social**

Plays with age appropriate toys

- Yes – 1
- No – 0
- .

Need for a home nurse or aid

- Yes – 0
- No – 1

Has your family structure changed as a result of the disease?

- Yes – 0
- No – 1

Has your financial situation changed as a result of the disease?

- Yes – 0
- No – 1

Has your work or employment changed as a result of the disease?

- Yes – 0
- No – 1

Have your social relationships changed as a result of the disease?

- Socialization with family or friends avoided because of disease-0

- Socialization with family or friends decreased because of disease-1
- No change in social relationships due to disease-2

Since having this child, have you felt that you cannot handle things very well?

- Never or almost never -2
- Most of the time-1
- All of the time-0

Since having this child, have you felt unable to do things that you enjoy doing?

- Never or almost never -2
- Most of the time-1
- All of the time-0

Since having this child, has your normal daily routine been impacted? Ex. mealtime, bedtime, etc.

- Never or almost never -2
- Most of the time-1
- All of the time-0

Do you feel your child is unable to participate in family activities?

- Never or almost never -2
- Most of the time-1
- All of the time-0

Overall, do you feel this disease has impacted your family's quality of life?

- Never or almost never -2
- Most of the time-1
- All of the time-0

**Motor Milestones:**

Reach with both arms

- Yes – 1
- No – 0

Roll over

- Yes – 1
- No – 0

Pull to stand

- Yes – 1
- No – 0

Cruise along furniture

- Yes – 1
- No – 0

**Gross Motor:**

- Walks independently – 4
- Walks with assistive device– 3
- Walks short distances but requires wheelchair-2
- Wheel Chair bound  – 1
- Bedridden – 0

**Fine Motor:**

- Dresses self – 2
- Dresses with Assistance – 1
- Needs full assistance – 0
